# Sex Differences in Thermogenesis Structure Behavior and Contact within Huddles of Infant Mice

**DOI:** 10.1371/journal.pone.0087405

**Published:** 2014-01-31

**Authors:** Christopher Harshaw, Jay J. Culligan, Jeffrey R. Alberts

**Affiliations:** Department of Psychological & Brain Sciences, Indiana University, Bloomington, Indiana, United States of America; University of Minnesota, United States of America

## Abstract

Brown adipose tissue (BAT) is a thermogenic effector abundant in most mammalian infants. For multiparous species such as rats and mice, the interscapular BAT deposit provides both an emergency “thermal blanket” and a target for nestmates seeking warmth, thereby increasing the cohesiveness of huddling groups. Sex differences in BAT regulation and thermogenesis have been documented in a number of species, including mice (*Mus musculus*)–with females generally exhibiting relative upregulation of BAT. It is nonetheless unknown whether this difference affects the behavioral dynamics occurring within huddles of infant rodents. We investigated sex differences in BAT thermogenesis and its relation to contact while huddling in eight-day-old C57BL/6 mouse pups using infrared thermography, scoring of contact, and causal modeling of the relation between interscapular temperature relative to other pups in the huddle (T_IS_^rel^) and contacts while huddling. We found that females were warmer than their male siblings during cold challenge, under conditions both in which pups were isolated and in which pups could actively huddle in groups of six (3 male, 3 female). This difference garnered females significantly more contacts from other pups than males during cold-induced huddling. Granger analyses revealed a significant negative feedback relationship between contacts with males and T_IS_^rel^ for females, and positive feedback between contacts with females and T_IS_^rel^ for males, indicating that male pups drained heat from female siblings while huddling. Significant sex assortment nonetheless occurred, such that females made more contacts with other females than expected by chance, apparently outcompeting males for access to each other. These results provide further evidence of enhanced BAT thermogenesis in female mice. Slight differences in BAT can significantly structure the behavioral dynamics occurring in huddles, resulting in differences in the quantity and quality of contacts obtained by the individuals therein, creating sex differences in behavioral interactions beginning in early infancy.

## Introduction

Small mammals such as rodents occupy thermal niches radically different from those of their larger mammalian relatives. House mice (*Mus musculus*), for example, have far more labile core body temperatures [1]–[3] and prefer warmer ambient conditions [4]–[6] than do humans. By huddling with conspecifics mice can nonetheless increase their metabolic efficiency by as much as 40–65% [7]–[10], increasing growth rates [11], [12] and reducing nutritive energy requirements [13]. Group-housed mice thus spend a great deal of time huddling under standard laboratory conditions [14], which are thermally more comfortable to humans than to mice [4]–[6]. Species across diverse taxa–from penguins to bats to rats and marmots–similarly rely upon social thermoregulation when facing varying degrees of cold [15]. Because huddling animals are, at once, producers and consumers of metabolic heat, the thermal and metabolic characteristics of a given species can, theoretically, significantly structure its social life (cf. [16]–[18]).

Heat is a particularly valuable commodity for mammals such as the rodents, given their small size, correspondingly high surface-to-mass ratio, and low thermal inertia [15], [19], [20]. This is even more true during early development, when yet smaller size, poorer insulation, and immature capacity for shivering render infants highly vulnerable to heat loss [21]. Most infant mammals nonetheless possess large quantities of thermogenic brown adipose tissue (BAT), with the largest deposits centered around the neck and interscapular region [22]–an ontogenetic adaptation that partially buffers infants from the limitations of altriciality [21]. In addition to BAT, many rodents are born into litters of up to ten or more pups, often reared communally with other litters (e.g., [23]), and thus spend a great deal of time huddling during early development [19], [24]. Such huddling has been shown to constitute an active group regulatory mechanism; a huddle can regulate its collective exposed surface area, expanding and contracting in response to increases and decreases in ambient temperature [25]–[27]. Despite such data and the importance of social thermoregulation in rodents generally, relatively little is known about the dynamics of behavior *within* huddles [3].

There is a growing appreciation that BAT thermogenesis is not just an emergency response, but a modulated regulator of infant thermal homeostasis [21]. As such, we suggest that it is also a phenotypic element, a variable but frequently present feature of an infant’s body that can modulate a pup’s *attractiveness* to other pups. By activating BAT a pup generates a potent thermal stimulus for nearby pups seeking warmth (cf. [28])–a target localized such that contact it elicits from others may be as protective of cardiac, thoracic, and neural functioning as BAT thermogenesis itself [29]–[31]. BAT thermogenesis should thus function to increase the cohesiveness of huddles [28], [32] and prior to the onset of olfactory-guided huddling [33], [34] group regulatory behavior via huddling should emerge from the combined influence of thermogenesis via BAT activation (i.e. each pup regulating its heat production/modulating its attractiveness to others) and thermotaxis (i.e. each pup moving toward warmth) [28], [32], [35]–[40].

BAT activation is indeed known to be critical for effective huddling during cold challenge [28], [32], [39]. Sokoloff et al. [32], for example, found that Syrian golden hamster (*Mesocricetus auratus*) pups–a species which has superior thermotactic abilities but lacks BAT thermogenesis [39]–huddle less effectively than do rat (*Rattus norvegicus*) pups, which possess BAT. Employing mixed weight-matched groups of rat and hamster pups, it was found that hamster pups tended to dominate contact with their thermoproductive rat huddlemates, likely due to their superior thermotactic ability [41]. In a study of rat pups, Sokoloff and Blumberg [28] found furthermore that pharmacological inactivation of BAT compromised huddling effectiveness, and that in mixed huddles, pups with active BAT tended to avoid contact pups with inactive BAT, preferentially huddling with each other. Such studies suggest that effective huddling does indeed emerge from the combined influence of BAT thermogenesis and thermotaxis, and that there is both competition and cooperation in groups of huddling pups (cf. [15], [28]), with the benefits of thermogenesis–or contribution to what might be viewed as a “thermal commons”–being shared *unevenly* rather than evenly [42] by members of the group [28].

Sex differences in BAT and its regulation have been reported in both rodents [43]–[45] and humans [46], [47]. Studies of adult rats and mice, for example, indicate that females have a higher threshold for BAT activation (i.e. activate BAT sooner upon cooling than males) [43] and that BAT is present in greater relative quantities [44], [45], [48]–[51] and has a higher thermogenic capacity [44], [45], [51], [52] in females. Nevertheless, little attention has been paid to the ontogeny of sex differences in BAT, and no studies to date have addressed whether sex differences in BAT affect interactions occurring between and among male and female pups in the huddle. In a recent study of C57BL/6 mice [27], we found that female pups were significantly warmer, on average, than their male siblings while both huddled together in response to a 20°C cold challenge on postnatal days 4 and 8 (PND4 and PND8)–a sex difference that has similarly been reported in adult mice housed under standard laboratory conditions (e.g., [48], [53], [54]).

Such findings raise the question of how sex differences in thermogenesis might structure interactions occurring within huddles of infant mice [55]–[57]. That is, do differences in thermogenesis result in differing experiences within the huddle during early development? More specifically: does the higher thermogenic output of females render them more attractive and thus garner them more contacts while huddling in mixed-sex groups? Here we replicate the finding of a thermal advantage for female C57BL/6 mouse pups over male siblings [27], in tests both within and outside the huddle, applying a combination of non-invasive infrared thermography, a novel method of scoring and analyzing contact behavior, and causal modeling of the behavioral thermodynamics of huddling pups. In particular, we were interested in whether there was a sex-dependent relationship between BAT activation and social contact (i.e. contact with other pups) in huddling groups, and if so, if evidence of either variable being causally dominant could be recovered from time series data obtained from groups of actively huddling pups. We demonstrate the utility of Granger analysis [58], [59] in the latter endeavor, revealing the presence of feedback relationships between contact and BAT thermogenesis during cold challenge that vary in sex-dependent manner.

## General Method

### Ethics Statement

All animal care and procedures were conducted in accordance with the guidelines of the National Institutes of Health Guide for the Care and Use of Laboratory Animals and the Bloomington Institutional Animal Care and Use Committee (BIACUC) at Indiana University (IU). All experiments described here were approved by the IU BIACUC (IU #12–024).

### Subjects

All animals were derived from C57BL/6J stock purchased from Jackson Labs (Bar Harbor, Maine) and bred in the Animal Behavior Laboratory at Indiana University. Litters were born and reared in standard mouse cages (30×13×19 cm) with food and water available *ad libitum*. The vivarium was maintained on 12∶12 h light/dark cycle (lights on at 0700 h) at 22±2°C. Postnatal day 8 (PND8; day of birth = PND 0) pups were employed because fur development impedes accurate thermography in the C57 strain after PND8.

### Procedure, Data Acquisition, etc

#### Apparatus/test environment

All tests were performed within a double-walled glass chamber (height = 30 cm; dia = 15.2 cm) on a round platform (dia = 11.25 cm), the surface of which was 21.5 cm from the chamber’s upper edge. The platform was constructed of 1.27 cm Styrofoam insulation (Dow Chemical Company), circled by polyethylene mesh (height = 15 cm) to prevent contact with the glass wall of the chamber, covered with a circular piece of clear plastic sheeting for easy cleaning between sessions. Air temperature within the chamber (Ta) was controlled by circulating chilled or heated water through its walls. An ICI 7320 P-Series infrared thermal imaging camera (Infrared Cameras Inc., Beaumont, TX) and Sony DXC-151A video camera were mounted above the testing chamber, such that both cameras could simultaneously capture images of pups at angles nearly perpendicular to the testing platform.

#### Temperature logging

Ambient air temperature (T_a_) was monitored continuously during both experiments and logged at 1 min intervals using a Type K thermocouple (located 1.5 cm above the platform) connected to an Omega HH802U thermometer and Omega Software for Windows, Ver. 1.6 (Omega Engineering, Inc., Stamford, CT), running on a Dell Latitude E6400 laptop.

#### Thermal imaging and video frame capture

Thermal images (Experiments 1 and 2) and video frame grabs (Experiment 2) were acquired simultaneously, once per minute, time-locked to the temperature logger. Thermal images were acquired via IR Flash ver. 2.0 for Windows (Infrared Cameras Inc., Beaumont, TX) running on a Dell Latitude E6400 laptop and video frame grabs acquired using a Sony DXC-151A video camera and Scion Image 1.62a, running on a Power Mac 64 (Mac OS 9.1).

### Data Analysis

#### Analysis of thermal images

**Figure 1:**
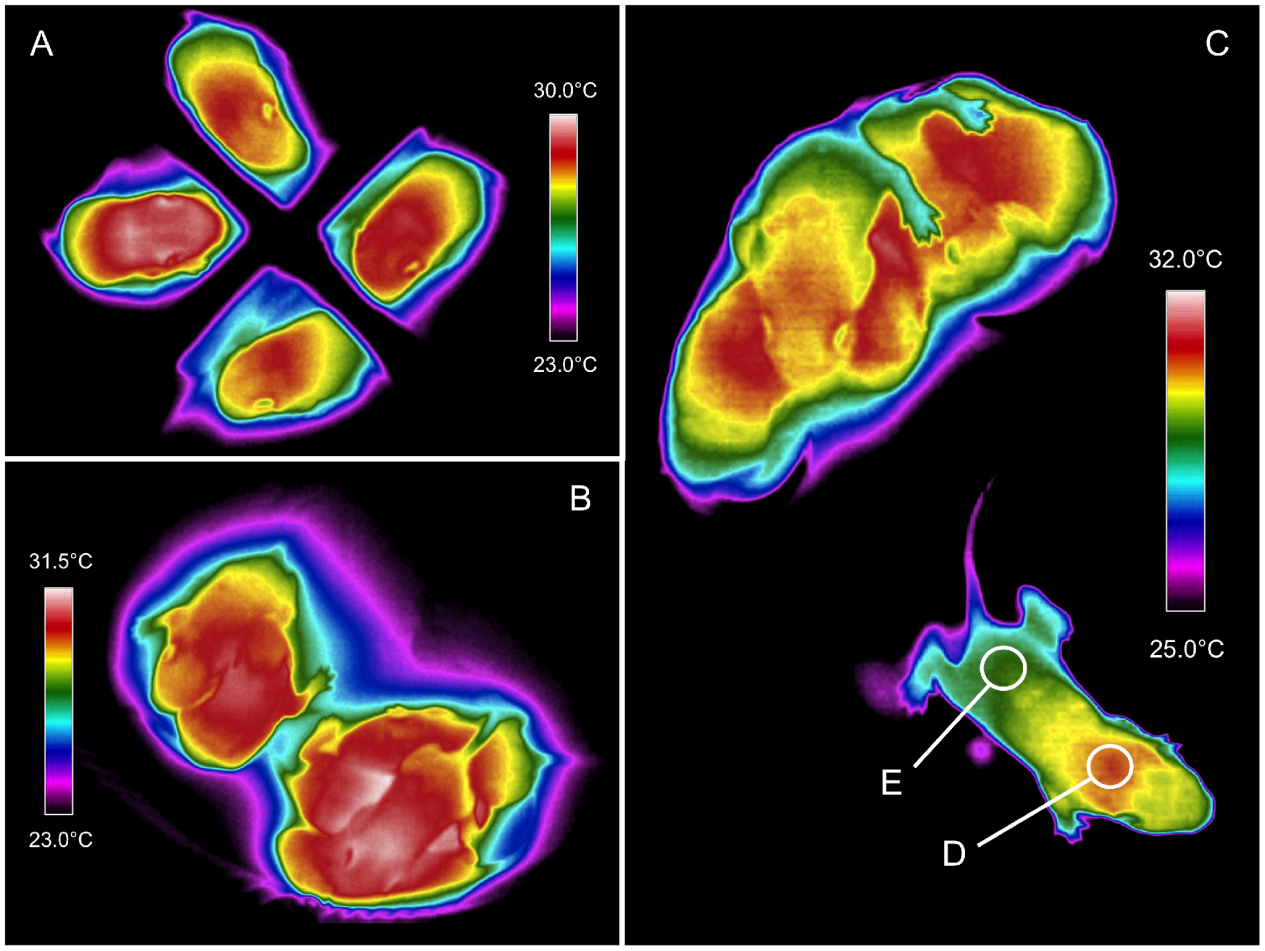
Infrared thermography. (**A**) Sample thermograph from Experiment 1, showing 2 male (top and left) and 2 female (bottom and right) pups during cold challenge. (**B–C**) Sample thermographs of litters during cold challenge in Experiment 2. In (**C**) the zones used for measuring interscapular (**D**) and rump (**E**) temperatures (T_IS_ and T_rump_, respectively) are shown for a pup that has separated from its huddle.

All thermal measurements were made by an experimenter blind to the sex of pups. From each thermograph, *body surface* temperatures from the interscapular (T_IS_) and/or rump (T_rump_) regions of each pup were obtained using ICI IR Flash. The interscapular (IS) region overlies the largest BAT deposit in the body and the temperature of the overlying skin increases when BAT is active (Fig. 1D). The pup’s rump contains no BAT and provides a baseline body surface temperature (Fig. 1E). The difference between these two regions (T_Δ_ = T_IS_ – T_rump_) is a validated and frequently used proxy for BAT activation [27], [28], [60]. Video frames were used to identify individual pups in corresponding thermal images and, whenever possible, both T_IS_ and T_rump_ were obtained for all pups. Figure 1D–E depicts how circular zones were superimposed on the regions of interest, centered on the body’s midline, providing an average temperature for all enclosed pixels. Zone diameters corresponded to an actual size of.55 cm or approximately half of the average body width of a PND8 pup. If only a pup’s IS or rump region was visible then measurement was obtained only for that region and no T_Δ_ was calculated for that pup at that time step. If a pup was lying on its side, was under the huddle or otherwise not visible, then no thermal measurements were obtained for that pup for that frame. Average T_IS_, T_rump_ and T_Δ_ measurements reported are thus averages of all available data for a given frame.

#### Statistical analysis

All statistics were calculated using *R*, version 2.15.1 (The R Foundation for Statistical Computing, Vienna, Austria) or IBM SPSS Statistics, version 20 (IBM Corp.), with a 5% criterion for significance (two-tailed). Sex differences in T_IS_, T_rump_, T_Δ_ (Experiments 1 and 2) and contact (Experiment 2) were evaluated using two complementary statistics. First, the *consistency* of sex difference was determined using Sign tests performed on time series of average values for each time step, with any missing data points (due to an inability to obtain measurements because of huddling, posture, etc.) for either sex excluded for the other sex for each litter. These tests were used instead of parametric repeated-measures analysis of variance (ANOVA) because the data in Experiment 2 violated the assumption of independence required for ANOVA, given that the two groups of interest (males and females) interacted continuously throughout the experiment. The same approach was adopted in Experiment 1 for simplicity/consistency of presentation. Next, the *directionality* and *magnitude* of difference was analyzed using paired-sample T-tests on average values for each pup, with each pup paired against the closest weight-matched opposite sex sibling, and missing values for either pup removed for the paired sibling. Only pairs of measurements obtained under identical T_a_ were thus included in between-sex analyses. For contact behavior in Experiment 2, these analyses were performed by sex (average number of contacts), type (same sex, opposite sex) and sub-type (male-male, female-female, male-female).

## Experiment 1: Sex Difference in Thermogenesis in C57BL/6 Pups

Previously, we found that female C57BL/6 mouse pups were significantly warmer than their male siblings while huddling in mixed-sex groups during a 50 min temperature cycle that ran from 36.5°C to 20°C back to 36.5°C [27]. In that study, the ambient temperature nonetheless changed continuously, while pups also interacted freely, so it was not possible to determine if the thermal advantage displayed by females resulted from a physiological or behavioral difference or synergy between the two. The first experiment here thus sought to replicate our previous study under more controlled conditions: pairs of male and female siblings were tested simultaneously under conditions that prevented contact and interaction, with fixed temperature phases rather than a temperature cycle. Based on prior observations indicating sex differences in thermoregulation and BAT in rodents (e.g., [44], [51]) and mice specifically (e.g., [48]) we expected female pups to be warmer than their male siblings during cold-challenge.

### Methods and Procedure

#### Subjects

In total, 64 mouse pups (32 male, 32 female) served as subjects, drawn from 18 unculled litters of 6 to 9 pups (mean = 7.0±.23) on PND8. Each trial consisted of two male and female sibling pairs (four pups total), weight-matched within.2 g (average difference = .006±.03 g). Average weights were 4.26±.07 g for females and 4.27±.08 g for males. Whenever possible, a male-female pair from one litter was tested simultaneously with a male-female pair from another litter (10 trials/12 litters). In all other cases two male-female pairs from a single litter were tested (6 trials/litters).

#### Procedure

A Plexiglas divider inserted into the chamber created a separate compartment for each pup, preventing pups from making contact during testing (see Figure 1A). Pups were carefully removed from their dam and checked for the presence of milk bands; only pups displaying such bands were selected for testing. The tails of pups were then color-marked by sex/litter with a non-toxic marker and placed within separate compartments upon the platform within the testing chamber, wherein the air temperature was stabilized at 34.5–36.5°C. Once all pups were in the chamber, they were provided 15 min to acclimate and regain body heat lost during transfer from the dam/nest [61].

#### Temperature sequence

Testing involved a two-phase temperature sequence. During the first phase, the initial warm air temperature (T_a_) of 34.5–36.5°C (mean: 35.4±.12°C) was maintained for a further 25 min. Data collection began after 15 min (to capture the last 10 min of the warm phase, as a baseline). Water baths were then switched, and T_a_ within the chamber cooled rapidly to 21–23.5°C (mean: 22.5±.36°C). The second phase began upon cooling and consisted of a further 50 min of data collection. Trials thus lasted 90 min, with 60 min of data collection (10 min warm, 50 min cool).

#### Statistical analysis

Sex differences in T_IS_, T_rump_, T_Δ_ were analyzed as described in the General Method, however the data were also separated into a Warm phase (first 10 min of the trial) and Cool phase (last 10 min of trial) for the purpose of comparing thermal measurements under the two conditions, particularly during the portion of the trial when BAT activation should be maximal.

### Results

**Figure 2:**
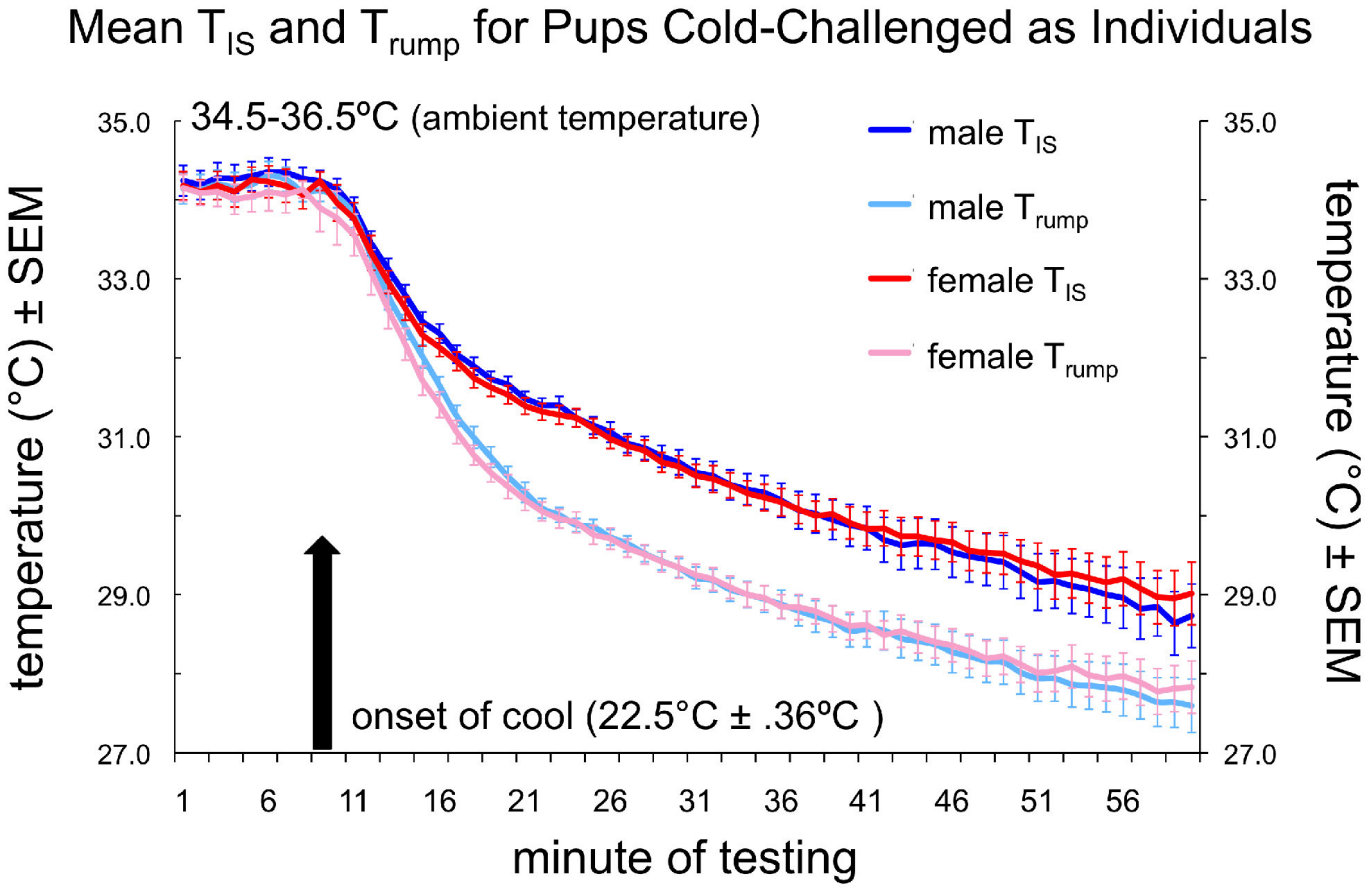
Thermal measurements for Experiment 1. Average temperatures for interscapular (T_IS_) and rump (T_rump_) regions ± SEM for male (blue lines) and female (red/pink lines) PND8 mouse pups.

As can be seen in Figure 2, there was little difference between male and female T_IS_ and T_rump_ measures until the latter portion of the cool phase. Sign tests comparing the full time series for T_IS_, T_rump_, and T_Δ_ revealed that females had consistently greater T_Δ_ values than males (Z = −2.16, *p*<.04), but no significant sex difference in absolute T_IS_ or T_rump_ measures (Z = −1.3, *p* = .19; Z = −.68, *p* = .51, respectively). If we restricted our analysis to the last half of the trial, in contrast, females showed consistently warmer T_IS_ (Z = −3.10, *p*<.003) and T_rump_ (Z = −4.09, *p*<.00005) measures, as well as greater T_Δ_ values (Z = −2.08, *p*<.04) than males. Paired sample t-tests on individual male-female pairs, however, failed to show a significant difference in T_IS_, T_rump_, or T_Δ_, although females trended toward a higher score than males on each of these measures during the cool phase (see Table 1).

**Table 1:** Thermal Measures in Experiment 1 by Sex.

average in °C (SEM) Paired sample T-tests						
measure		males	females	t score	*p*-value	effectsize(dz)
**Warm phase**						
**(first 10 min)**	T_IS_	34.09 (.15)	34.10 (.14)	.26	.400	.046
	T_rump_	33.95 (.17)	33.99 (.17)	1.01	.161	.184
	T_Δ_	0.12 (.03)	0.09 (.04)	−1.16	.128	.224
**Cool phase**						
**(last 10 min)**	T_IS_	28.93 (.28)	29.19 (.23)	1.31	.100	.235
	T_rump_	27.74 (.23)	27.94 (.19)	1.27	.107	.189
	T_Δ_	1.18 (.06)	1.25 (.04)	1.21	.119	.240

*Note*. Results for Paired sample t-tests (one-tailed) on thermal measures obtained under identical T_a_ for male-female sibling pairs, under conditions in which pups were isolated and thus could not interact. Female measures were, on average, higher than male measures, however, these failed to reach statistical significance. SEM = Standard error from the mean.

In summary, male-female sibling pairs showed no difference in thermal measures during the warm phase, but females trended toward warmer T_IS_ and T_rump_ scores and greater T_Δ_ values than males during the cool phase. The females’ trend toward being significantly warmer emerged during the latter portion of the trial, when BAT activation was maximal–as indicated by T_Δ_ values. We took this result as further evidence of enhanced BAT thermogenesis in female mouse pups [27]–albeit of a potentially small effect size that would likely require a larger number of subjects and/or more prolonged cold-challenge to achieve statistical significance. Nevertheless, statistical significance is not a necessary indicator of real-world or biological significance, and we were ultimately interested in the question of whether this sex difference in thermogenesis impacts behavioral dynamics within huddles of infant mice.

## Experiment 2: Sex Differences in Thermogenesis and Contact Behavior During Huddling

Experiment 2 addressed the question of whether sex differences in thermogenesis are associated with differences in contact behavior within mixed-sex huddles of PND8 mouse pups. That is, if females are more thermogenic, do they receive a greater quantity or different quality of contact while huddling? Presumably, huddling in a cool environment emerges from the combined influence of BAT thermogenesis and thermotactic responses–each pup striving to make contact with the warmest spot in the huddle. Thus, we hypothesized that the greater heat output of females during cold challenge would garner them more contacts while huddling in mixed-sex groups than male siblings. We also hypothesized that there would be differing competitive dynamics between male and female pups during cold challenge, as have been documented in mixed huddles of BAT inactivated and BAT activated pups [28], with females showing more contacts with each other than would be expected by chance. In contrast, we expected to find no relationship between BAT thermogenesis and contact during periods when pups faced no thermal challenge.

### Methods and Procedure

#### Subjects

In total, 48 PND8 mouse pups (24 male, 24 female) served as subjects, selected from unculled litters of 6 to 9 pups (8 litters total).

#### Apparatus/test environment

All tests were performed within the same double-walled glass chamber used in Experiment 1. However, no divider was used and pups could thus freely interact within the chamber.

#### Procedure

Pups were carefully removed from their dam and checked for the presence of milk bands; only pups displaying such bands were selected for testing. Pups were then individually weighed, marked with a water-based paint, and placed upon the platform within the testing chamber, where the air temperature was stabilized at 34–36°C. Once all pups were in the chamber, they were provided 15 min to acclimate and regain body heat lost during transfer from the dam/nest [61], prior to the onset of data collection.

#### Temperature sequence

Testing involved a standardized temperature sequence consisting of a warm and cool phase. During the warm phase, the initial warm air temperature (T_a_) of 34–36°C (mean: 35.1±.2°C) was maintained for a further 45 min. Water baths were then switched, and T_a_ within the chamber rapidly cooled to 20–22°C (mean: 21.1±.2°C). The cool phase consisted of a further 45 min of data collection, initiated once T_a_ reached 23°C. Trials thus lasted 111–112 min (15 min acclimation +45 min warm +6–7 min cool down +45 min cool).

### Data Analysis

#### Contact behavior (contactogons)

**Figure 3:**
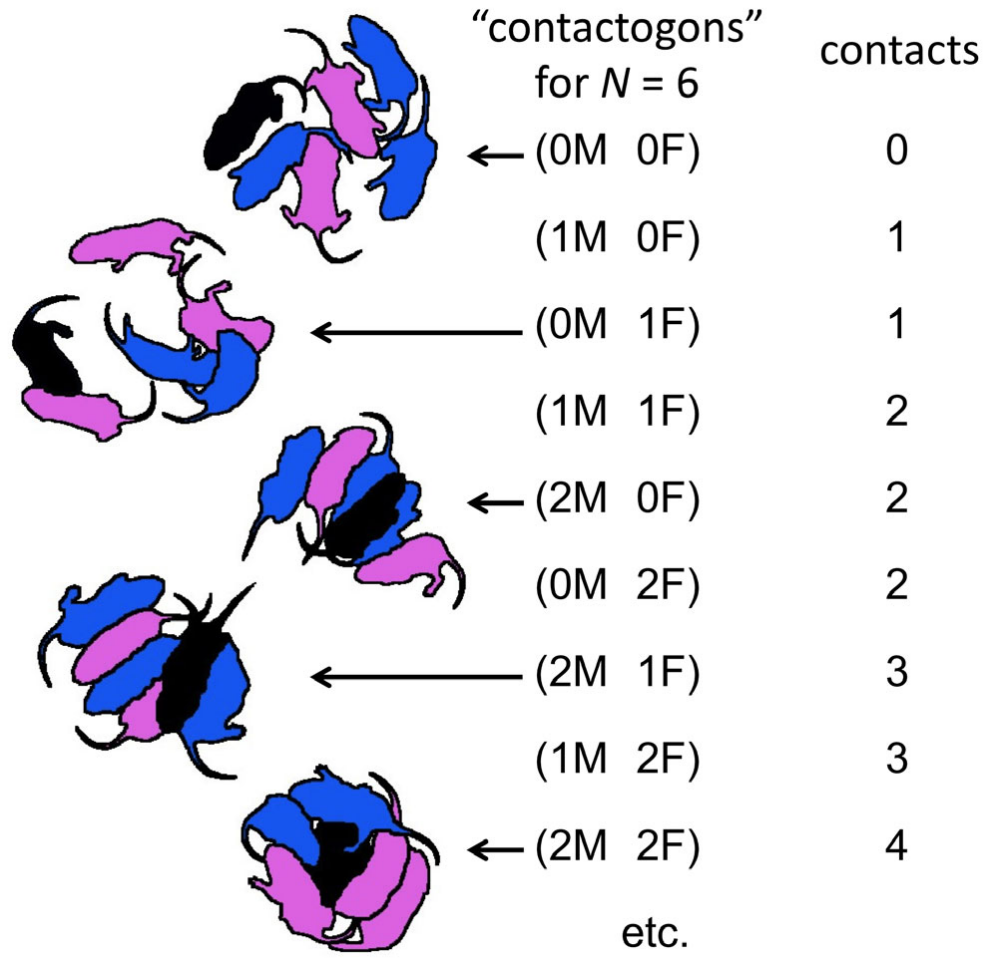
Scoring of contact behavior. A depiction of the system used for scoring contact behavior, adapted from that employed by Sokoloff and Blumberg [28]. Each pup in the litter was scored each minute for how many males (blue pups) and females (pink pups) they were in contact with, excluding contacts via tails and outstretched paws. Each combination of contacts was assigned a unique identifier or “contactogon”. For example, 0 M 2 F designates contact with zero males and two females. Contactogons possible for only a single sex (e.g., 3 M 0 F) are not shown, and were collapsed into a single category for the purposes of statistical analysis.

We employed a modified version of the scoring system used by Sokoloff and Blumberg [28], which involved a simple count of the number of pups with which a focal pup was in contact at each of a sequence of time points. With our modification, illustrated in Figure 3, each pup in the huddle was scored at each time step for the number of male and female pups with which it was in contact, excluding contacts made via tails or outstretched paws. A pup scored as 0 M 0 F, for example, was not in contact with any other pups, whereas 2 M 3 F indicated contact with two male and three female pups (i.e. all of the other pups in the group). The scoring of specific contact patterns or *contactogons*, permitted the analysis of relationships between T_IS_ and overall number of contacts, contacts by sex, as well as whether or not the overall distribution of contactogons differed from that expected by chance.

#### Analysis of thermal images

As in Experiment 1, an experimenter blind to the sex of pups measured T_IS_ and T_rump_ for all pups in the huddle whenever possible (see Figures 1B and 1C). Because Experiment 2 was focused on sex differences in thermoregulation and the relationship between thermogenesis and contact rather than on BAT activity itself, our primary measure for correlational and causal analyses (described below) was a pup’s T_IS_ relative to its huddlemates or *relative T_IS_* (T_IS_^rel^). A pup’s relative T_IS_ was calculated by subtracting the average temperature of its IS region from the average T_IS_ for the group (T_IS_^rel^ = T_IS_^pup^ – T_IS_^group^) at each time point. The measure thus provides a better indicator of the relative attractiveness of a pup over the course of a trial than either T_IS_ or T_Δ_, while permitting between- and within-litter comparisons independent of T_a_.

#### Statistical analysis

To determine whether the distribution of contact types or contactogons between-sex deviated from that expected by chance, G-tests of goodness of fit (see [62]) were performed for each temperature phase. The G-test is similar to the chi-square goodness of fit test, but the G statistic is superior to the chi-squared in several respects (see [63]), including how well it approximates the chi-squared distribution [64]. Expected values were generated by multiplying the observed number of occurrences of each contact type for males and females by the ratio of simple probabilities for that contactogon for males and females. For example, 0 M 0 F, 1 M 1 F, and 2 M 2 F are equally likely for males and females, giving a 1∶1 ratio if contacts are made at random, whereas the probability of 2 M 0 F would be.1 for males and.3 for females, giving a 1∶3 ratio. All contact types displayable by only a single sex (e.g., 3 M 2 F) were collapsed into a single category. In the case of a significant G-test (indicating that the *overall* distribution of contactogons differed from that expected by chance), post-hoc Fisher’s exact tests were performed for each contactogon, with a Bonferroni correction for multiple tests (*α* = .05/10 = .005), to determine which of these differed from chance. To examine the relationship between contact and pup thermal status and whether that relationship varied by sex, we calculated Pearson product-moment correlations for average values of T_IS_^rel^ and total number of contacts as well as T_IS_^rel^ and contacts with males and females. Similar analyses were performed for the relationship between pup weight relative to huddlemates (weight^rel^ = weight^pup^ – weight^group^), contact variables, and T_IS_^rel^. All such correlations were evaluated using a Bonferroni correction for multiple tests (*α* = .05/3 = .0167).

#### Granger analyses

To examine the causal dynamics between contact and thermal status, we performed a series of Granger analyses [58], [59]. Granger analysis relies upon a statistical rather than philosophical definition of causality, specifying that causes must precede effects, and that a variable *Y* can be considered to cause another variable *X* if and only if *Y* contains information that is useful in forecasting future values of *X* significantly better than a model including only past values of *X* [65]. Granger analyses examining the relationships between the time series for T_IS_^rel^ and total contacts, contacts with males, and contacts with females were performed, with a Bonferroni correction for multiple tests (*α* = .05/6 = .0083). These were accomplished by (a) averaging all male and female pups within-litter to produce an average times series for male/female T_IS_^rel^, male/female contacts with males, and male/female contacts with females, (b) combining each litter time series into a single series, with blanks inserted between litters to prevent cross-contamination at the edges of the series, and (c) calculating Granger statistics for the resulting master time series. Although Granger analysis can lead to spurious conclusions under certain conditions, it is used here under circumstances where a causal relationship between the two variables of interest (contact and thermal status) can be safely assumed (see [65]), to determine in which direction causality is primarily running (i.e. contact→T_IS_^rel^ versus T_IS_^rel^→contact). Given that our design allowed direct comparison of Granger analyses on time series of equal length obtained on the exact same set of interacting individuals under two conditions–one where causal interaction is predicted (cool phase) and one where it is not (warm phase)–we interpret the obtained results as being strongly suggestive of causality. Whether or not one accepts a “causal” interpretation of these tests (Granger-cause is often used as a substitute), they demonstrate, at a minimum, temporal precedence in change and that one time-series contains information that is useful in forecasting another [59].

### Results

#### Thermal and contact analyses

**Figure 4:**
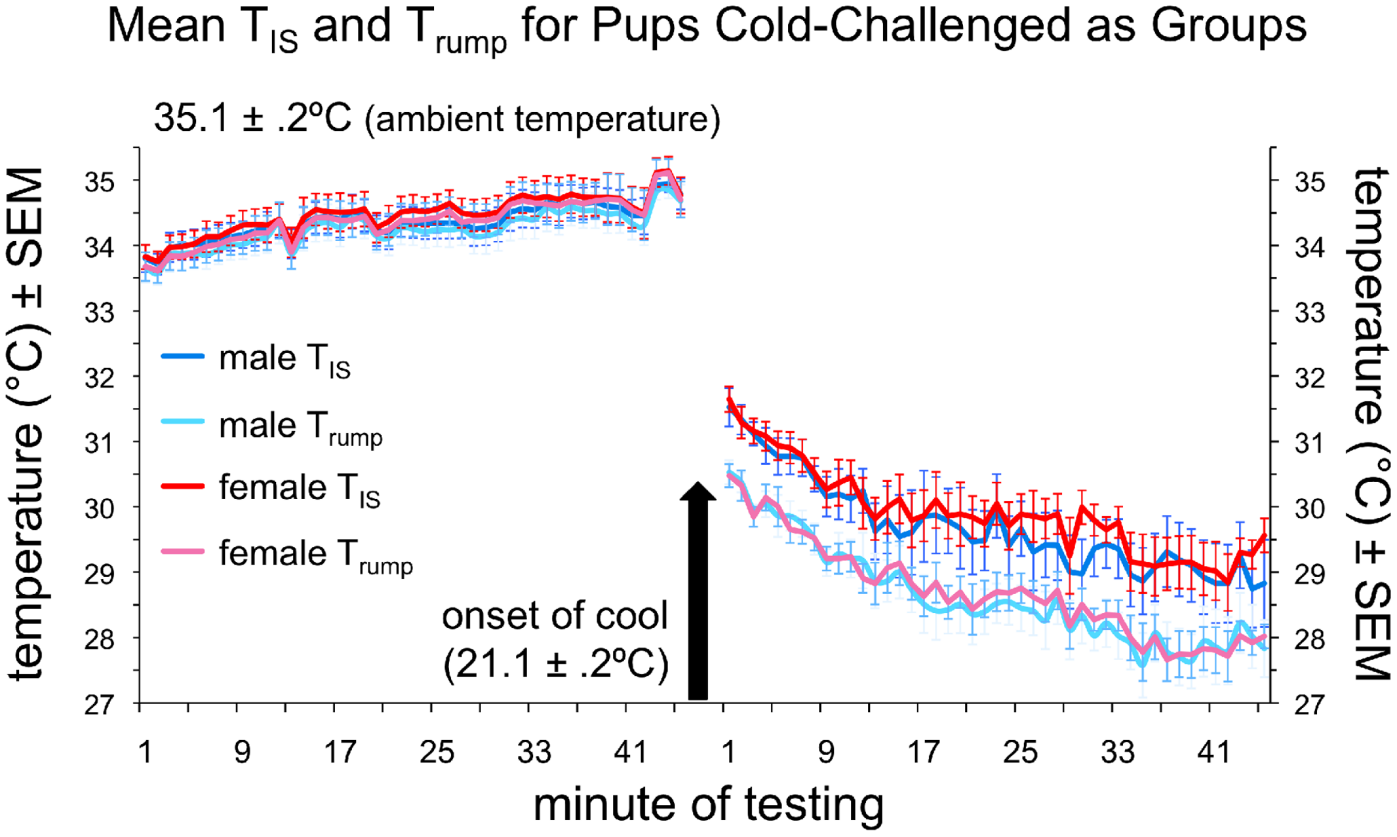
Thermal measurements for Experiment 2. Average temperatures for interscapular (T_IS_) and rump (T_rump_) regions ± SEM for male (blue lines) and female (red/pink lines) PND8 mouse pups.

As shown in Figure 4, female pups had consistently higher interscapular and rump temperatures than their male huddlemates during both the warm and cool phases of the experiment. Although relatively small, the difference (.11±.01°C for T_IS_ during warm; .21±.03°C for T_IS_ during cool) was significant for T_IS_ and T_rump_ measures during both phases of the experiment and for T_Δ_ during the cool phase (see Table 2). Paired sample t-tests on male-female sibling pairs produced a similar pattern of results: female T_IS_ and T_rump_ were significantly warmer than male T_IS_ and T_rump_ during the warm phase (t = −2.13, *p*<.05, effect size *dz* = .477, and t = −2.23, *p*<.04, effect size *dz* = .497, respectively) and female T_Δ_ was significantly higher than male T_Δ_ during the cool phase (t = −2.20, *p*<.04, effect size *dz* = .447), indicating greater BAT thermogenesis in females during cold challenge.

**Table 2:** Consistency of Sex Differences in Thermal and Contact Measures in Experiment 2.

Sign Tests			
measure		Z score	*p*-value(<)
**Warm phase**			
	T_IS_	−5.71*	.000001
	T_rump_	−5.67*	.000001
	T_Δ_	−.50	.617
	Total Contacts	−.68	.499
	Same/Opposite Sex	−.18	.855
	Male-Male/Fem-Fem	−.83	.405
**Cool phase**			
	T_IS_	−5.37*	.000001
	T_rump_	−2.09*	.05
	T_Δ_	−4.67*	.000005
	Total Contacts	−4.37*	.00002
	Same/Opposite Sex	−3.04*	.003
	Male-Male/Fem-Fem	−4.06*	.00005

*Note*. Results for Sign tests on thermal and contact measures obtained under identical T_a_ for the two sexes. *Asterisks* indicate a significant difference. In all cases of a significant difference this indicated higher female over male values and greater same- versus opposite-sex and female-female over male-male contacts.

To rule out alternative explanations for the observed sex difference in body surface temperatures, analyses of weight and order of placement into the testing apparatus were performed. There was neither a significant difference in order of marking (t = 1.6, *p* = .124, effect size *d* = .290) nor a difference in weight (t = −1.65, *p* = .113, effect size *d* = .329); males were in fact slightly heavier (4.27±.08 g) than females (4.15±.08 g). During the warm phase, there was no overall correlation between weight^rel^ and T_IS_^rel^ (*r* = −.113, *p* = .45), although there were apparent trends toward opposite correlations between the same variables for males (*r* = −.36, *p* = .091) and females (*r* = .305, *p* = .148). During the cool phase, in contrast, there was a trend toward a significant positive correlation between weight^rel^ and T_IS_^rel^ overall (*r* = .312, *p*<.04)–a relationship that was statistically significant for males (*r* = .495, *p*<.015) but not females (*r* = .332, *p* = .113). Taken together, these results indicate that the thermal advantage displayed by females could not have been driven by a weight difference between the sexes or by the correlation between weight^rel^ and T_IS_^rel^.

**Figure 5:**
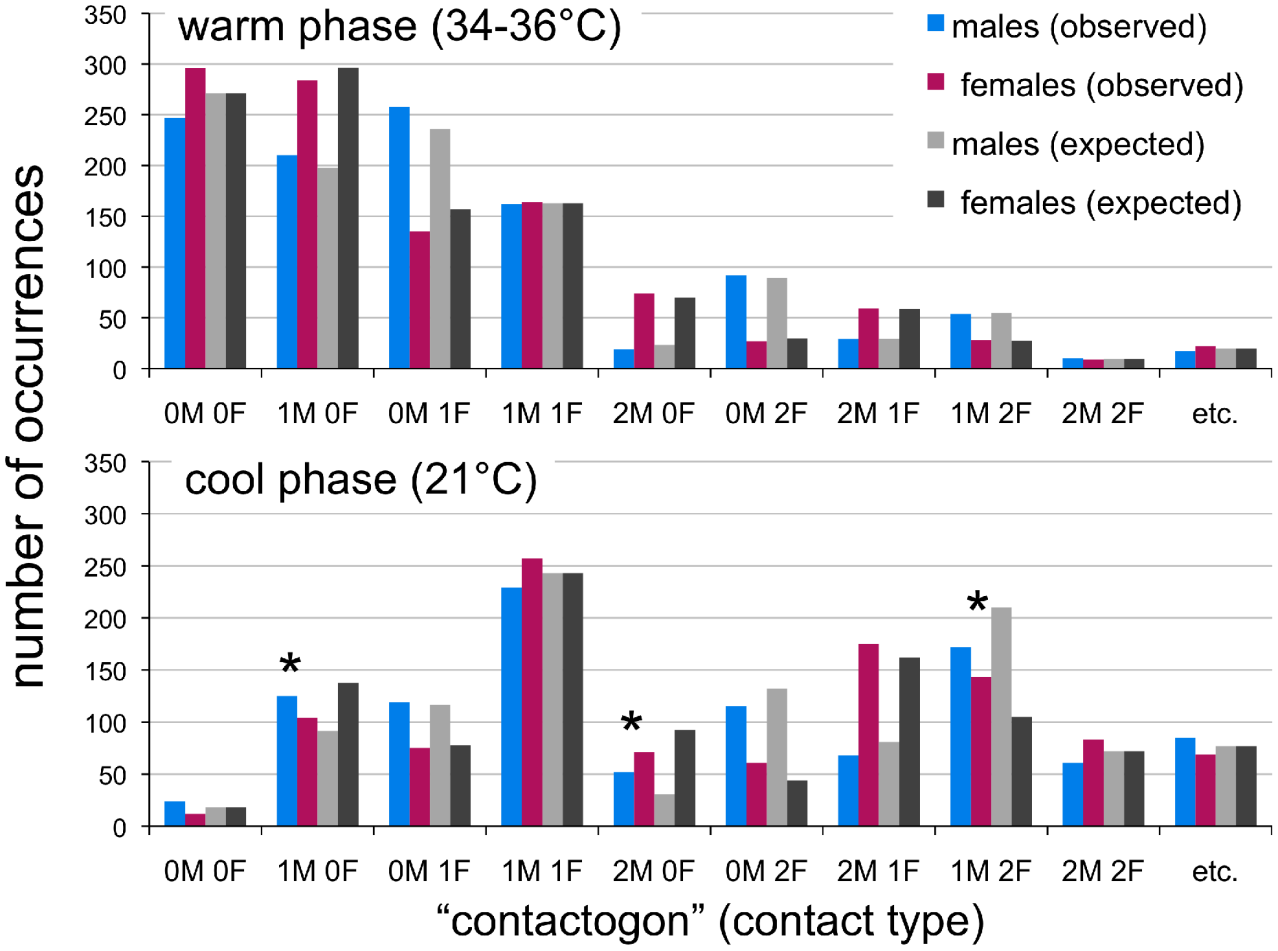
Contactogon distributions for warm and cool phases. Distribution of contactogons (contact types) during the warm and cool phases of Experiment 2 (upper and lower panels, respectively). Values on the *y*-axis indicate total number of occurrences of each contactogon observed and expected by chance for males and females during the 50 minute trial. As can be seen, the distribution is skewed leftward, to more dispersed contactogons during the warm phase (which did not differ significantly from the chance distribution; *G* = 6.3, *p* = .705), and rightward, toward greater amounts of contact during the cool phase (which differed significantly from the distribution expected by chance; *G* = 39.7, *p*<.00001). Asterisks indicate significant deviation from chance responding in specific contactogons using Fisher’s exact tests (*p*<.005).

**Figure 6:**
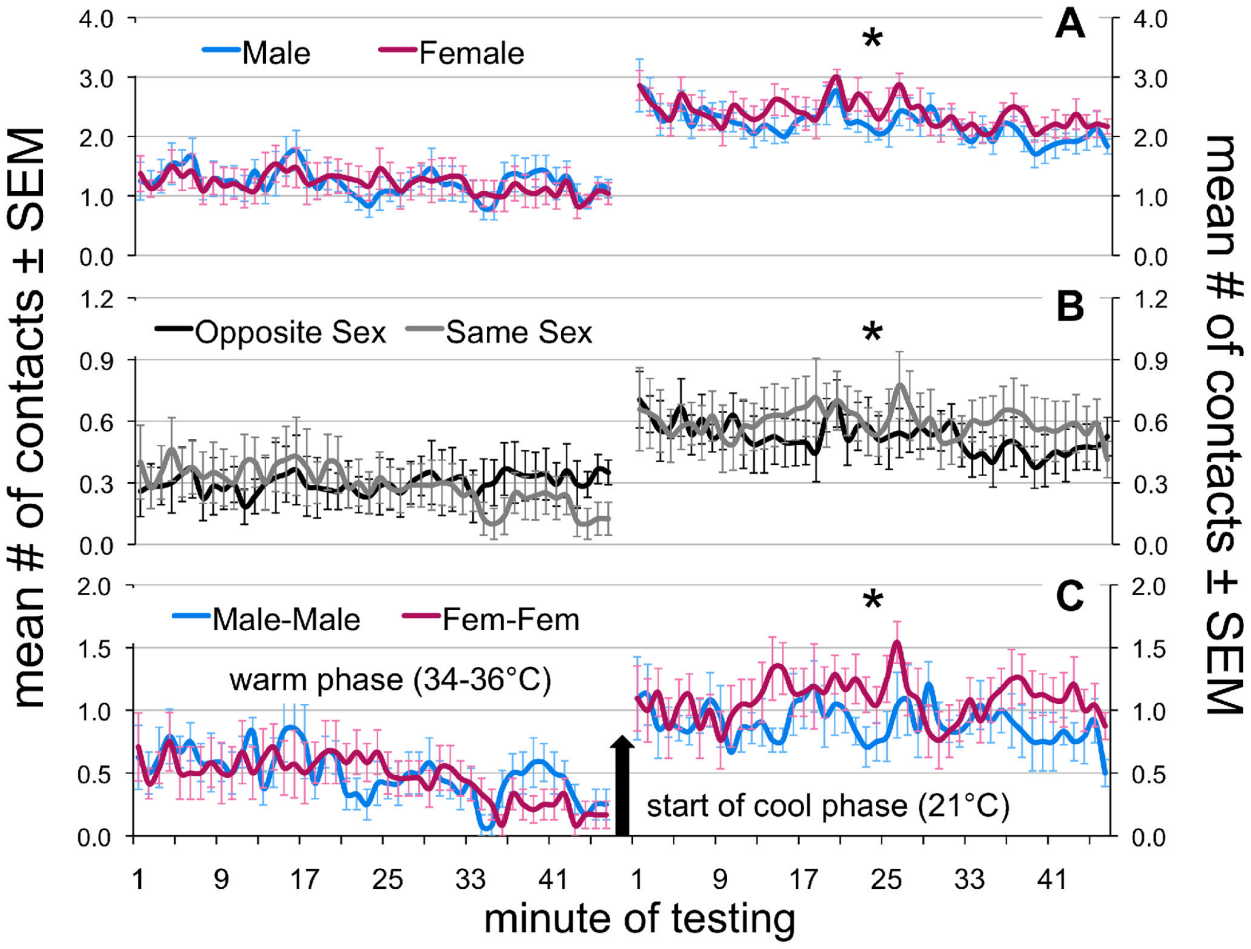
Contact time series for warm and cool phases. Average number of contacts over time ± SEM, by (**A**) sex, (**B**) type (opposite- vs. same-sex), and (**C**) sub-type (male-male vs. female-female). Values in (**B**) are corrected for the fact that opposite-sex are always slightly more likely than same-sex contacts in a 2∶3 ratio (opposite-sex contacts x.4; same-sex contacts x.6). Asterisks indicate a systematic difference (i.e. a non-random distribution of differences) between the two series using a Sign test (*p*<.005).

Analysis of pup contact patterns at warm temperatures revealed no deviations from chance responding (G test of goodness of fit, G = 6.34, df = 9, *p* = .705, effect size *w* = .08; Fig. 5). That is, there were no differences in total contacts for males and females, same- and opposite-sex contacts, or number of male-male (MM) compared to female-female (FF) contacts (see Table 2; Fig. 6). As can be seen in the gradual divergence between same- and opposite-sex contacts in Figure 6B, however, there were significantly more opposite- than same-sex contacts during the second half of the warm phase (sign test, *p*<.0009). During cold challenge, in contrast, contact patterns shifted such that female pups garnered significantly more contacts than males in the same huddles, while there were also significantly more same- than opposite-sex contacts, as well as more FF than MM contacts (see Table 2; Fig. 6). Accordingly, the overall between-sex distribution of contact patterns also differed significantly from that expected by chance (G = 39.71, df = 9, *p*<.00001, effect size *w* = .23). Specifically, this included more 1 M 0 F and 2 M 0 F displayed by males (Fisher’s exact tests, *p*<.004, effect sizes *g* = .13 and.18, respectively) and more 1 M 2 F displayed by females (Fisher’s exact test, *p*<.002, effect size *g* = .12) than would be expected by chance (see Fig. 5). As a whole, these results indicate significant sex assortment within huddles of infant mice during cold challenge.

#### Relationship between contact, thermal status, and weight

**Figure 7:**
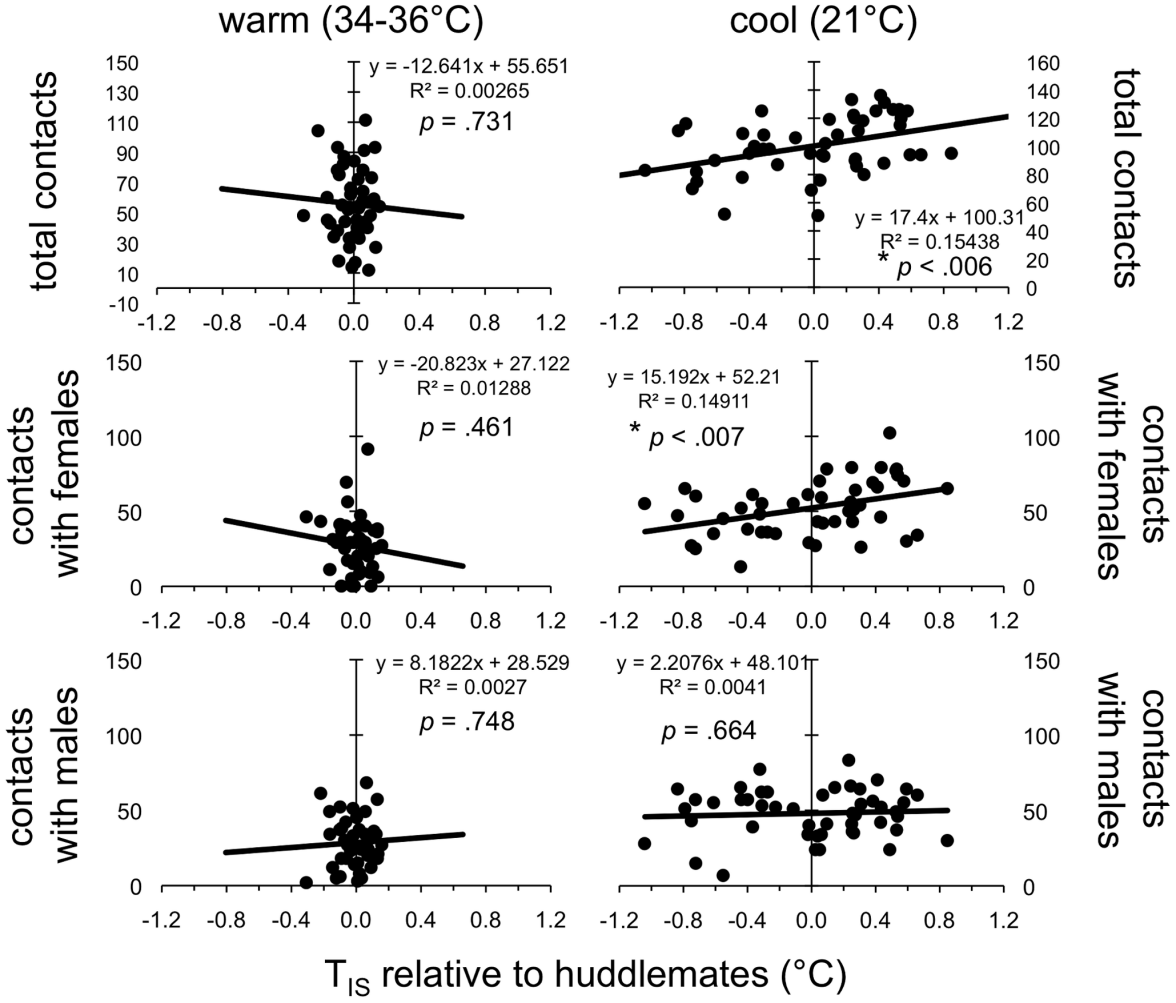
Correlation between relative T_IS_ and contact. Linear regressions, coefficients of determination (*R^2^*), and *p*-values for Pearson product moment correlations on T_IS_ relative to huddlemates (T_IS_^rel^) and (a) total contacts, (b) contacts with females, and (c) contacts with males, during the warm and cool phases of Experiment 2 (left and right, respectively). Asterisks indicate significant correlation, using a criterion of *α* = .05/6 = 0083. As can be seen, there was no relationship between T_IS_^rel^ and contact during the warm phase, and a significant correlation between T_IS_^rel^ and both total contacts and contacts with females, but not contacts with males, during the cool phase.

As shown in the graphs on the left side of Figure 7, there was no relationship between pup thermal status (T_IS_^rel^) and contact behavior during the warm phase. During the cool phase, in contrast, a significant correlation between the total contacts a pup received and its average T_IS_^rel^ emerged (*r* = .39, *p*<.006). The correlation between pup weight^rel^ and total contacts, in contrast, was not significant (*r* = .228, *p* = .119). When contacts with males and females were analyzed separately, the correlation with T_IS_^rel^ held for contacts with females (*r* = .39, *p*<.007) but not for contacts with males (*r* = .06, *p* = .664). Similarly, the correlation with weight^rel^ was significant for contacts with females (*r = *.313, *p*<.04), but not for contact with males (*r* = −.065, *p* = .666). These results indicate both a stronger influence of T_IS_^rel^ than weight^rel^ on the contacts a pup receives and a significant interaction between thermal status and contact, mediated by the sex of the pup being contacted.

**Figure 8:**
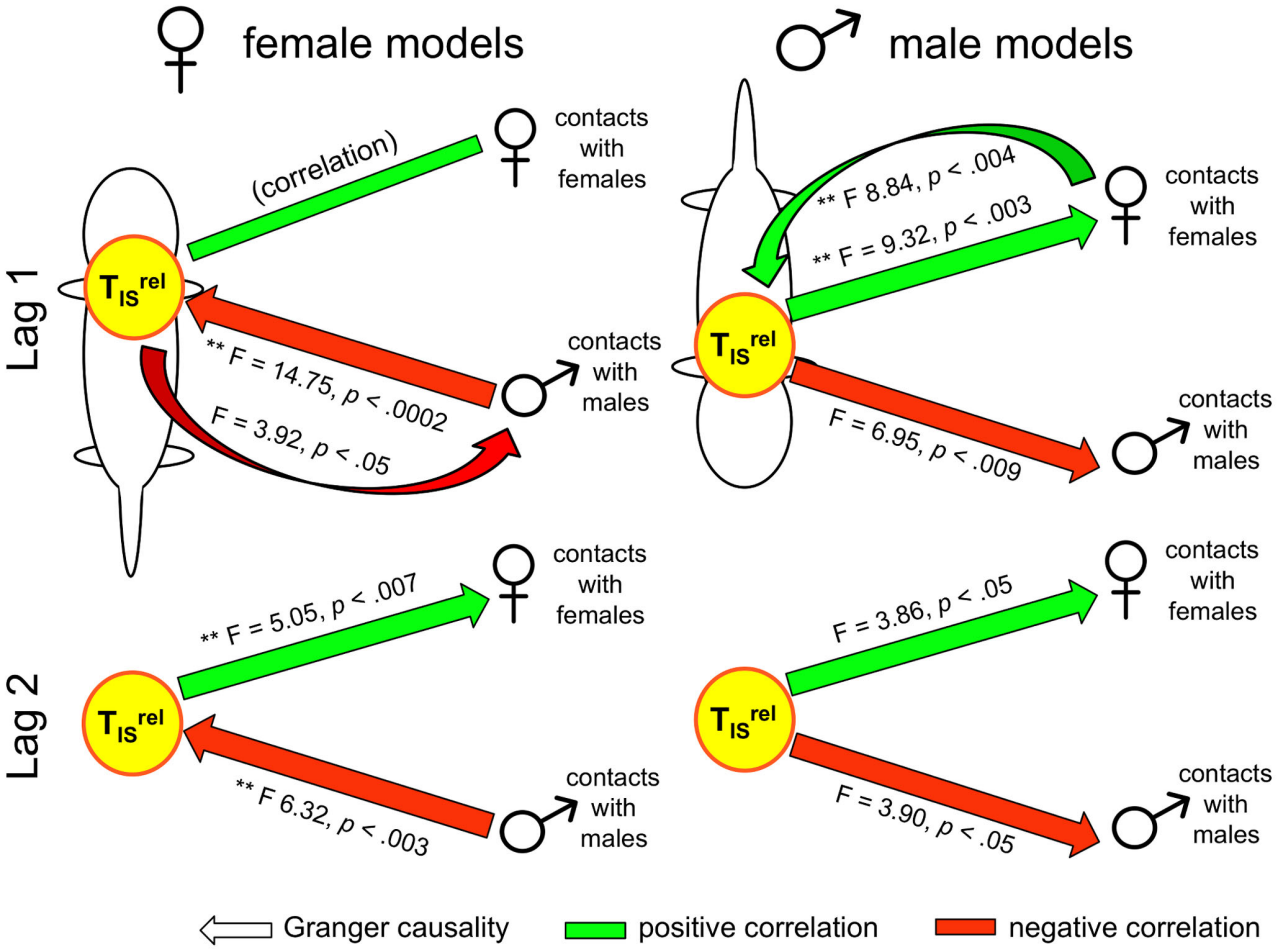
Granger (causal) analyses of relative T_IS_ and contact during cold challenge. The results of Granger analyses on relative T_IS_ (T_IS_^rel^) and contact with males and females. Asterisks indicate significant Granger causality, evaluated at *α* = .05/6 = .0083. Non-significant results (p<.05) are also shown to depict trends in the data. Arrows indicate that a change in one variable at time *t_n_* predicts a change in another variable at *t_n+lag_*. For example, in all but the Female Lag 0 models, T_IS_^rel^ is a stronger predictor of contacts with females than the reverse. Arrows in both directions indicate a feedback relationship between the two variables [65]; for example, our results indicate negative feedback between female T_IS_^rel^ and female contacts with males and positive feedback between the male T_IS_^rel^ and male contacts with females. The coloration of arrows indicates significant lagged Pearson product moment correlations between the two variables (*p*<.001), with positive and negative correlations indicated by green and red, respectively. Tests between T_IS_^rel^ and total contacts were run, but are not shown given that none were statistically significant. Models for Lag 3 were constructed but are not shown. For females, the model was null (no significant Granger causality detected), whereas for males the only significant effect was that male T_IS_^rel^ Granger caused male contacts with males (F = 4.94, *p*<.003).

**Figure 9:**
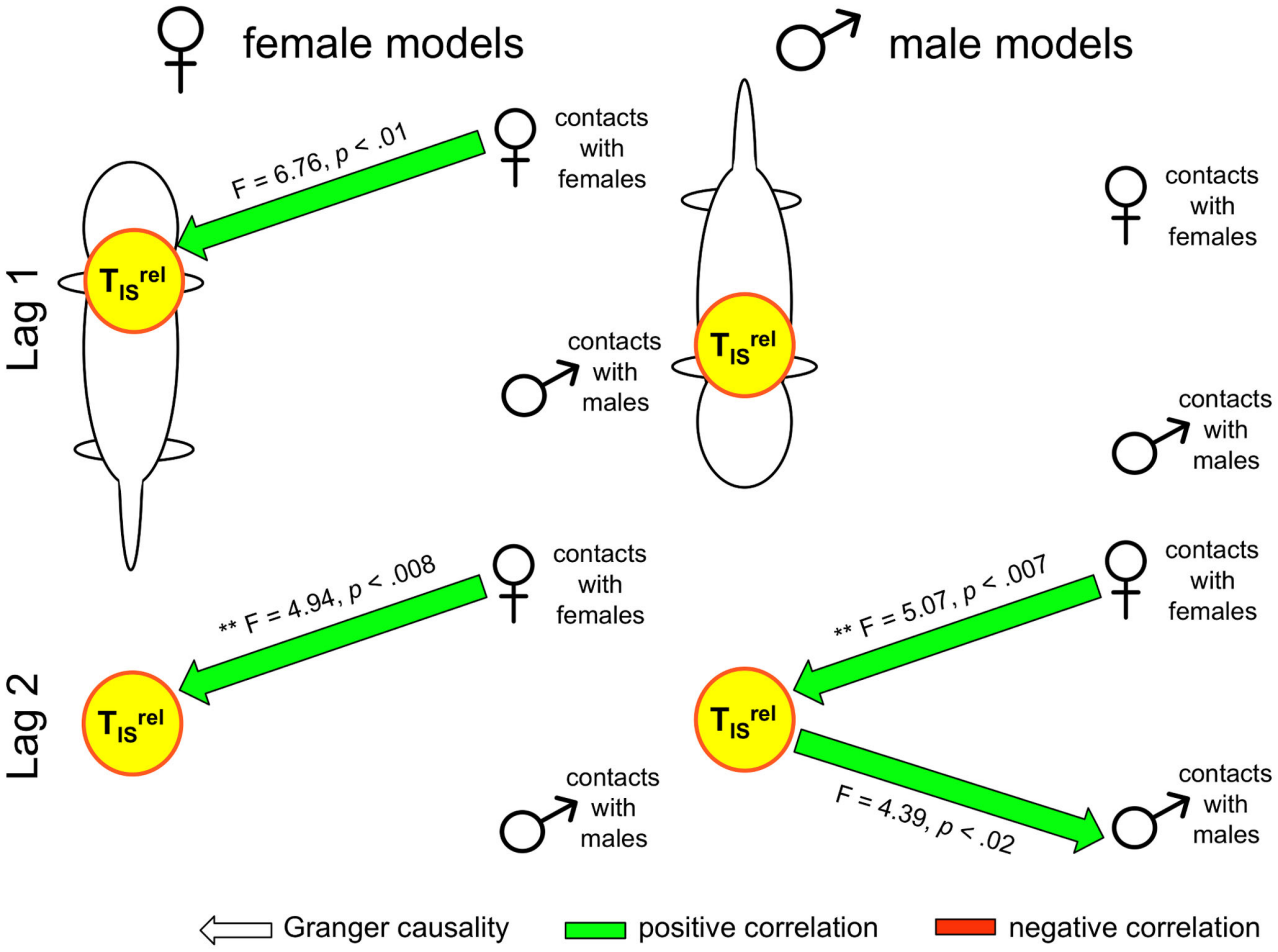
Granger (causal) analyses of relative T_IS_ and contact during the warm phase. The results of Granger analyses on relative T_IS_ (T_IS_^rel^) and contact with males and females. Asterisks indicate significant Granger causality, evaluated at *α* = .05/6 = .0083. Non-significant results (p<.05) are also shown to depict trends in the data. The coloration of arrows indicates significant lagged Pearson product moment correlations between the two variables (*p*<.05), with positive and negative correlations indicated by green and red, respectively. Tests between T_IS_^rel^ and total contacts were run, but are not shown.

Figure 8 depicts, for males (right) and females (left), the results of Granger and lagged-correlational analyses of T_IS_^rel^, contacts with males, and contacts with females. Consistent with the results of our correlational analyses, there were a number of interesting differences between the causal models for males and females. At Lag 1, the most salient difference was a negative feedback relationship between contacts with males and T_IS_^rel^ for females, and a positive feedback relationship between contacts with females and T_IS_^rel^ for males (see [65]). At Lag 2, T_IS_^rel^ was found to Granger-cause contacts with females (positively) for both sexes, whereas the relationship between T_IS_^rel^ and contacts with males was consistently negative, but varied in direction depending on sex (i.e. contacts with males predicted T_IS_^rel^ for females and T_IS_^rel^ predicted contacts with males for males). During the warm phase of the experiment, in contrast, there were no statistically significant relationships found between T_IS_^rel^, contacts with males, and contacts with females at Lag 1 (see Fig. 9). For females there was, however, a significant relationship between *total* contacts and T_IS_^rel^ (F = 7.21, *p*<.008), with more total contacts predicting greater T_IS_^rel^ (*r^2^* = .066, p<.0001). At Lag 2, in contrast, contact with females Granger-caused T_IS_^rel^ for both males (F = 5.07, p<.007) and females (F = 4.94, p<.008), with directionality being ambiguous for males (*r^2^* = .005, p = .263) and contact with females predicting greater T_IS_^rel^ for females (*r^2^* = .089, p<.0001).

#### Summary

In summary, we found that female PND8 mouse pups had significantly warmer T_IS_ and T_rump_ regions than did their male siblings during both the warm and cool phases of the experiment. In addition, T_Δ_ values were significantly higher for females than for males during the cool phase, indicating greater BAT thermogenesis in females during cold challenge [27], [28], [60]. Although the results of the present experiment appear to indicate a larger difference between the sexes than was found in Experiment 1, it seems likely that behavioral interactions between male and female pups (not present in Experiment 1) may have amplified physiological differences between the sexes. In support of this argument, correlational analyses revealed a significant positive correlation between T_IS_^rel^ and contacts with females but no relationship between T_IS_^rel^ and contacts with males during cold challenge (Fig. 7). Granger analyses of the same time series during the cool phase moreover revealed a negative feedback relationship between contacts with males and T_IS_^rel^ for females, with contacts with males tending to diminish female T_IS_^rel^. At the same time, there was a positive feedback relationship between contacts with females and T_IS_^rel^ for males, with contacts with females being a net thermal benefit to males. Our analysis of contactogons suggests that males were nonetheless frequently outcompeted for contact with females, given that there was significant sex assortment during cooling and significantly more FF than MM contacts (Fig. 5).

During the warm phase there was no correlation between contact and T_IS_^rel^. Granger analyses moreover revealed no relationship between T_IS_^rel^, contacts with males, and contacts with females at Lag 1, for either sex. Lag 2 analyses nonetheless revealed a significant positive relationship between T_IS_^rel^ and contacts with females for both sexes. Given that pups were relatively inactive during the warm phase and contactogon distributions revealed no significant deviations from chance responding (Fig. 4), it seem likely that the latter finding is the result of a passive transference of heat from females, due to incidental contacts. It was observed on several occasions, anecdotally, that at some ambient temperatures within the range of 34–36°C all of the males tended to be quiescent and all of the females active, whereas at other temperatures all of the females tended to be quiescent and all of the males active. The fact that there were fewer same- than opposite-sex contacts during the second half of the warm phase than would be expected by chance would be explained by sex differences in temperature-dependent sleep or activity.

## Discussion

The present study demonstrates that sex difference in thermogenesis can significantly affect the behavioral interactions occurring within huddles of infant C57BL/6 mice. In tests of both isolated and huddling pups, we confirmed that female pups are warmer, on average, than their male siblings [27]. This was particularly true during cold challenge, presumably due to enhanced brown adipose tissue (BAT) thermogenesis in females [43]–[45]. In Experiment 1, physiological response to cold challenge was tested in male-female sibling pairs under conditions in which pup weights were matched closely, T_a_ was identical, and pups were prevented from making physical contact. Under these carefully controlled conditions, female pups trended toward being significantly warmer than their male siblings when cold challenged, particularly at the end of the trial, when BAT activation–as indicated by T_Δ_ values–was maximal (Fig. 2; Table 1). Greater thermogenesis in female pups was confirmed in Experiment 2, and found to be statistically significant under conditions in which males and females could actively huddle in response to cold challenge and thus interact (Fig. 4; Table 2). The females’ higher temperatures appeared to make them more attractive huddling targets, as females garnered significantly more contacts than their male siblings during cooling (Fig. 6B).

Interestingly, this sex difference in thermogenesis also gave rise to spontaneous sex assortment, as female pups displayed more contact with female siblings and male pups contacted male siblings more than was expected by chance (Figs. 5, 6C). The most parsimonious explanation for this finding is that female pups had greater access to each other because any two randomly selected females would have, on average, been more attracted to each other than to any randomly selected male–an asymmetry of attraction that when iterated across time and consecutive competitive interactions would result in assortment. This finding suggests that *homophily* or the clustering of similar individuals in groups or networks [66] can be driven solely by regulatory similarities among individuals, a fact that may partially explain some instances of sex- and reproductive-status-based homophily in the wild, such as occurs in bat roosts [67]–[69]. Such dynamics are also relevant to the *time-budget hypothesis* of sex assortment, which argues that in sexually dimorphic species, sex differences in body size can influence activity synchronization (e.g., the timing of eating, drinking, and rest), which can encourage segregation by sex in mixed-sex groups [70], [71]. The time-budget hypothesis implicitly assumes that regulatory similarities between individuals can drive their assortment. It is important to emphasize that such homophily can emerge purely from simple taxic responses (e.g., thermotaxis, chemotaxis) in a non-uniform environment and thus does not require complex cognitive input, control, or “choice.”

Our findings also indicate significant sex differences in behavioral dynamics within huddles of infant mice. First, correlational analyses revealed that the relationship between relative T_IS_ (T_IS_^rel^)–an indicator of how attractive a pup should be to its huddlemates when huddlemates are motivated to contact warmth–varied depending both on ambient temperature and the sex of contact being made. That is, there was no relationship between T_IS_^rel^ and contacts during the warm phase, whereas there was a significant positive correlation between T_IS_^rel^ and contacts with females, but not between T_IS_^rel^ and contacts with males during the cool phase (Fig. 7). Granger analyses performed on time series for T_IS_^rel^, total contacts, contacts with males, and contacts with females helped to illuminate this difference, revealing significant differences in the interactional dynamics occurring between the sexes. That is, for female pups there was a negative feedback relationship between their T_IS_^rel^ and contacts with males, whereas for male pups there was an opposite, positive feedback relationship between T_IS_^rel^ and contacts with females (Fig. 8). That such causal modeling reflects the dynamics occurring within the huddle is supported by the finding of no significant Lag 1 Granger causality between the same variables, for the same animals during the warm phase (Fig. 9).

There are a number of ways to view these findings. For example, from a behavioral ecological or game theoretic perspective *competition* between the sexes might be highlighted. Females appear to be overproducing and males under-producing or “cheating” slightly–“skimping on their share of the heating bill” [42]. Based on findings that genes of paternal origin tend to inhibit and genes of maternal origin tend to enhance capacity for BAT thermogenesis, Haig has argued that there is inter-genomic conflict between males and females, visible in genes regulating BAT [42], [72], [73]. The conflict occurs, in Haig’s view, because BAT thermogenesis within groups of huddling pups lends itself to exploitation, given that any heat contributed by an individual pup becomes a resource shared by all. In litters of mixed paternity, in particular, pups will share more maternal than paternal DNA, and it is in the interests of the mother to upregulate thermogenesis in her offspring and in the interests of the father to downregulate thermogenesis in his offspring, allowing them to invest more in growth [42], [72], [73]. Haig’s argument nonetheless rests on the assumption that *the benefits of thermogenesis are shared equally* within a huddling group and thus that strategic underproduction can be advantageous. As demonstrated here, however, there are emergent costs to such underproduction, given that pockets of regulatory homophily can emerge in huddles with uneven thermogenic contribution. That is, assuming equal distribution of thermotactic responses [41], any two overproducers will be both more attractive and more attracted to each other, and thus have competitive advantage for accessing each other, while underproducers will tend to be outcompeted for such access [28].

From an ethological and lifespan developmental perspective, in contrast, *cooperation* between the sexes would be highlighted. That is, male and female rodents often have dissimilar reproductive roles, and in many cases it benefits males to be larger and more dominant than their competitors [74]. This may be particularly true of male mice, which are highly territorial and highly aggressive toward other males [75]–[77], with social dominance within a deme yielding overwhelming reproductive success [78]. Although the relation between body size and dominance status is equivocal under artificial group-rearing conditions [79], size is an important determinant of aggression and fighting in mice [18], [80], and fighting is important in achieving dominance and gaining territory [81], [82]. Given that BAT thermogenesis utilizes resources otherwise available for growth [83], [84], males may not be “cheating” by underproducing heat early on so much as “saving” energy to invest in growth that will be beneficial later in development. An optimal “strategy” for a male, under this view, may thus be to produce enough heat to be attractive to others in the huddle–enough to not be left completely ‘out in the cold’–while nonetheless making less than the mean thermogenic contribution for the group.

The present study was limited to pups of a single age (i.e. PND8), with huddling and BAT thermogenesis examined in a non-naturalistic context. It might be argued that the flat surface of our test chamber permitted artificially high degrees of freedom to huddling pups or otherwise amplified differences likely to be found under more naturalistic circumstances. For example, pups are usually located in a nest tended by one or more dams [85], and a high quality, dome-shaped nest or burrow will tend to trap heat contributed by individual pups, resulting in a warm, relatively homogenous microclimate [86]. The quality of nests constructed by dams nonetheless varies within and between strains (e.g., [87]), as does the quality of maternal care in general, including time spent on and off the nest [88], [89]. Nest temperatures also fall rapidly during periods of parental absence [90], [91]. Within our laboratory, C57BL/6 dams often build bowl- rather than dome-shaped nests and dams with PND8–9 pups have been observed to spend large amounts of time away from the nest during a 24 hr period, in bouts of up to 45–130 min (average: 10–25 min; [92]). A study in which the temperatures of cotton-wool nests containing C57 pups of various ages were measured moreover found nest temperatures to be markedly heterogeneous, with differences of up to 10°C within nests [90]. Taken together, it is thus likely that C57BL/6 litters experience periods of significant cooling under standard laboratory conditions, creating thermal conditions that at least approximate those of the present study. It should nonetheless be emphasized that C57BL/6 mice are an inbred laboratory strain, and future studies should examine sex differences in BAT thermogenesis and contact while huddling in different strains and under more naturalistic conditions.

The potential long-term consequences of early differences in metabolic, thermal, and contact-related phenotypes in mice also warrants attention (cf. [18]). In the present study, pup weight relative to other pups in the huddle (weight^rel^) was not significantly correlated with the number of contacts a pup received while huddling; the only significant correlation with weight^rel^, across sexes, was a positive correlation with contacts with females. In both rabbits and rats, heavier pups have been found to occupy more central positions in the nest, to exert less effort in huddling, and to be warmer than their littermates [57], [93]. Such advantageous positioning during the first postnatal week has moreover been found to correlate with greater fearfulness and less “proactivity” later in development (e.g., exploration, longer latency to jump from a cold shelf) in rabbits [55], [56]. Although the trend in the present study suggests a slight advantage for heavier mouse pups when huddling, relative thermal status (T_IS_^rel^) proved to be a stronger predictor of a pup’s position in the huddle (using contacts as a proxy) and weight^rel^ correlated with T_IS_^rel^ only for males. Whether the benefits of being heavier eventually override those of being warmer at some point in development for huddling mouse pups remains an open question. It is also unknown what if any long-term consequence attracting a greater quality or quantity of contacts early in development may have for mice, although the present study suggests a number of hypotheses deserving of exploration.

An additional question of interest is the downward, epigenetic influence of group-level variables such as litter size and sex ratio (i.e. litter composition) on BAT thermogenesis and contact behavior displayed by individual pups. For example, litter size has been found to affect both general activity level and several components of huddling behavior in rabbit pups [57] as well as adult anxiety- and aggression-related phenotypes in a number of mammals [94]–[96]. Litter sex ratio also affects a number of individual behavioral and morphological characteristics, via both prenatal [97], [98] and postnatal [99]–[101] influences. For example, differential exposure to sex hormones during embryonic development due to variable intrauterine positioning (e.g., being adjacent to two males versus two females) affects a number of sex-linked morphological, neural, and behavioral phenotypes of adult rodents and other mammals (see [97], [98]). Sex steroids directly affect BAT regulation [43], [45], [102], [103], so it is possible that litter sex ratio and intrauterine position may have a canalizing (i.e. feminizing or masculinizing) influence on the thermogenic and/or metabolic phenotypes displayed by individual pups. Such reorganization could, for example, mediate the effects of early steroid exposure on nest construction phenotypes in adult mice [104], [105].

In summary, the present study demonstrates that sex-specific variation in an individual phenotype–the amount of heat produced via BAT thermogenesis–can significantly impact contact and behavioral interactions between the sexes in groups of huddling C57BL/6 mouse pups. The advantages of viewing BAT as a modulator of pup attractiveness and of scaling individual thermogenesis with reference to group mean thermogenesis (i.e. employing relative interscapular temperature) for correlational and causal analyses were also demonstrated. The potential for observing large differences in behavioral dynamics stemming from small regulatory differences between individuals, as the consequences of those differences accumulate across time in an interacting group, was also emphasized. Although much remains unknown about the dynamics of behavior occurring within huddles, the methods introduced here suggest new avenues for exploring a number of important mechanistic and developmental questions regarding the regulatory and social behavior of rodents.
